# Toward Automation of Motor Assessments in Stroke: Proof-of-Concept Model to Interpret Balance and Gait in Patients With Stroke

**DOI:** 10.1016/j.arrct.2026.100673

**Published:** 2026-07-12

**Authors:** Yee Mah, Vassilios Tahtis, Abigail Beddard, Aryan Esfandiari, Jorge Cardoso, Parashkev Nachev

**Affiliations:** a School of Biomedical Engineering and Imaging Sciences, King’s College London, London, United Kingdom.; b King’s College Hospital, London, United Kingdom; c Queen Square Institute of Neurology, University College London, London, United Kingdom

**Keywords:** Artificial intelligence, Gait, Motion analysis, Rehabilitation, Stroke

## Abstract

**Objective:**

To evaluate the feasibility of automated action recognition and movement characterization during the Performance-Oriented Mobility Assessment (POMA) using markerless three-dimensional pose estimation and to explore whether data-driven whole-body movement analysis differentiates healthy adults from stroke survivors.

**Design:**

Observational, cross-sectional proof-of-concept study

**Setting:**

Single-center study conducted at a UK National Health Service Hospital stroke unit.

**Participants:**

Fifty-six healthy adults and 17 stroke survivors able to complete the POMA assessment (N=73).

**Intervention:**

Participants completed the POMA under therapist supervision while recorded using 2 synchronized RGB cameras. Major joints were extracted to generate skeletal representations, which were analyzed using machine-learning methods to automatically detect distinct POMA actions.

**Main Outcome Measures:**

Primary outcomes were overall action recognition accuracy and F1 scores for individual POMA actions. Secondary outcomes included qualitative and quantitative differences in movement patterns between healthy participants and participants with stroke derived from whole-body kinematic embeddings.

**Results:**

The action recognition model achieved a mean classification accuracy of 78.9%. Performance was highest for sitting, standing, and turning, with F1 scores of 0.95, 0.82, and 0.80, respectively. Low-dimensional embeddings of movement trajectories revealed distinct clustering patterns and greater dispersion among participants stroke, consistent with impaired balance and gait control.

**Conclusions:**

Markerless pose estimation combined with machine-learning analysis can accurately recognize actions and detect clinically meaningful differences in movement during a standardized balance and gait assessment. Although exploratory, these findings illustrate the potential of artificial intelligence to characterize movement patterns and identify kinematic features distinguishing patient groups. This approach offers a pathway toward scalable, objective, and automated motor assessment, with the potential to identify patients at risk of functional decline and support targeted clinical interventions.

Stroke remains a leading cause of adult disability worldwide, with motor impairment highly prevalent. Evidence indicates that substantial training dose and intensity are required to achieve clinically meaningful recovery.^1^^,^^2^ Accurate and consistent measurement of motor change is therefore critical, because without reliable outcomes, behavioral restitution cannot be distinguished from compensation.^3^ Consequently, clinicians are limited in their ability to detect change, plan intervention, and optimize rehabilitation. Outcome measurement underpins effective rehabilitation by providing an objective framework for evaluating progress. However, in clinical practice, outcome measures are frequently omitted or inconsistently applied, lack sensitivity, and are susceptible to subjectivity and inter-rater variability.^4^, ^5^, ^6^, ^7^, ^8^ Although robust and clinically meaningful motor outcome measures are well established^9^^,^^10^ and widely validated in populations with stroke, their reliance on trained assessors completing item-based ordinal scales renders them time consuming and difficult to implement amid competing clinical demands.^4^^,^^5^

Recent advances in computer vision present a potential paradigm shift in clinical movement analysis. Pose estimation algorithms enable markerless tracking of human movement from video data,^11^^,^^12^ allowing objective, fine-grained quantification of motor behavior that may exceed traditional observational methods. These approaches could enable automated capture of patient movement and interaction, supporting continuous assessment of motor performance and early identification of patients at risk of adverse events such as falls,^13^^,^^14^ aspiration,^15^ or shoulder subluxation.^16^ Because these systems evolve through exposure to data, they offer opportunities for automated clinical documentation and decision support, with substantial implications for rehabilitation practice.

The Tinetti Performance-Oriented Mobility Assessment (POMA) was originally developed to assess gait, balance, and fall risk in older adults^17^ and has since been validated across neurologic conditions, including Parkinson disease,^18^ multiple sclerosis,^19^ spinal cord injury,^20^ and stroke.^21^ As with other motor outcome measures, the POMA seeks to distill clinical expertise and visual judgment into a reproducible score reflecting patient performance. For complex motor tasks, segmentation into discrete components helps guide attention and reduce perceptual bias.

Given that the POMA incorporates key constructs recommended for evaluating lower-limb motor function, balance, and mobility after stroke,^10^ it was identified as a suitable candidate for automation. Current pose estimation models achieve joint localization accuracy of approximately 40-80 mm.^12^ By parameterizing the body as a three-dimensional (3D) joint-based skeleton over time, these models enable the derivation of kinematic features such as joint angles, velocity, symmetry, and coordination. Such descriptors provide a foundation for data-driven analyses of motor performance and the development of novel clinicometrics that may capture clinically relevant features overlooked by conventional scales.

This study describes the development of an artificial intelligence (AI) model to analyze pose estimation data from healthy participants and stroke survivors performing the POMA. Rather than replicating predefined scoring criteria, the model was trained to cluster whole-body movement trajectories based on interjoint dynamics. This approach provides insight into differences in balance and gait between healthy populations and populations with stroke, as well as within-group variability among participants with stroke that may reflect distinct impairments or compensatory strategies. It represents an early step toward data-driven identification of salient movement features that may hold clinical relevance.

The framework comprises the following 3 core components required for automated motor assessment: pose estimation, action recognition, and movement evaluation. Pose estimation reduces the body to a skeletal representation of key joints (fig 1). This work focuses on action recognition and movement evaluation, examining how AI methods can cluster movement patterns and extract kinematic features that differentiate groups. Using a dataset of participants without stroke assessed on the POMA by trained clinicians (Motion Analysis-Hospital Oriented Therapy Assessments in Stroke (MA-HOTAS)) alongside participants with stroke, the system was trained to identify POMA-related actions such as sitting, standing, and turning.

**Fig 1:**
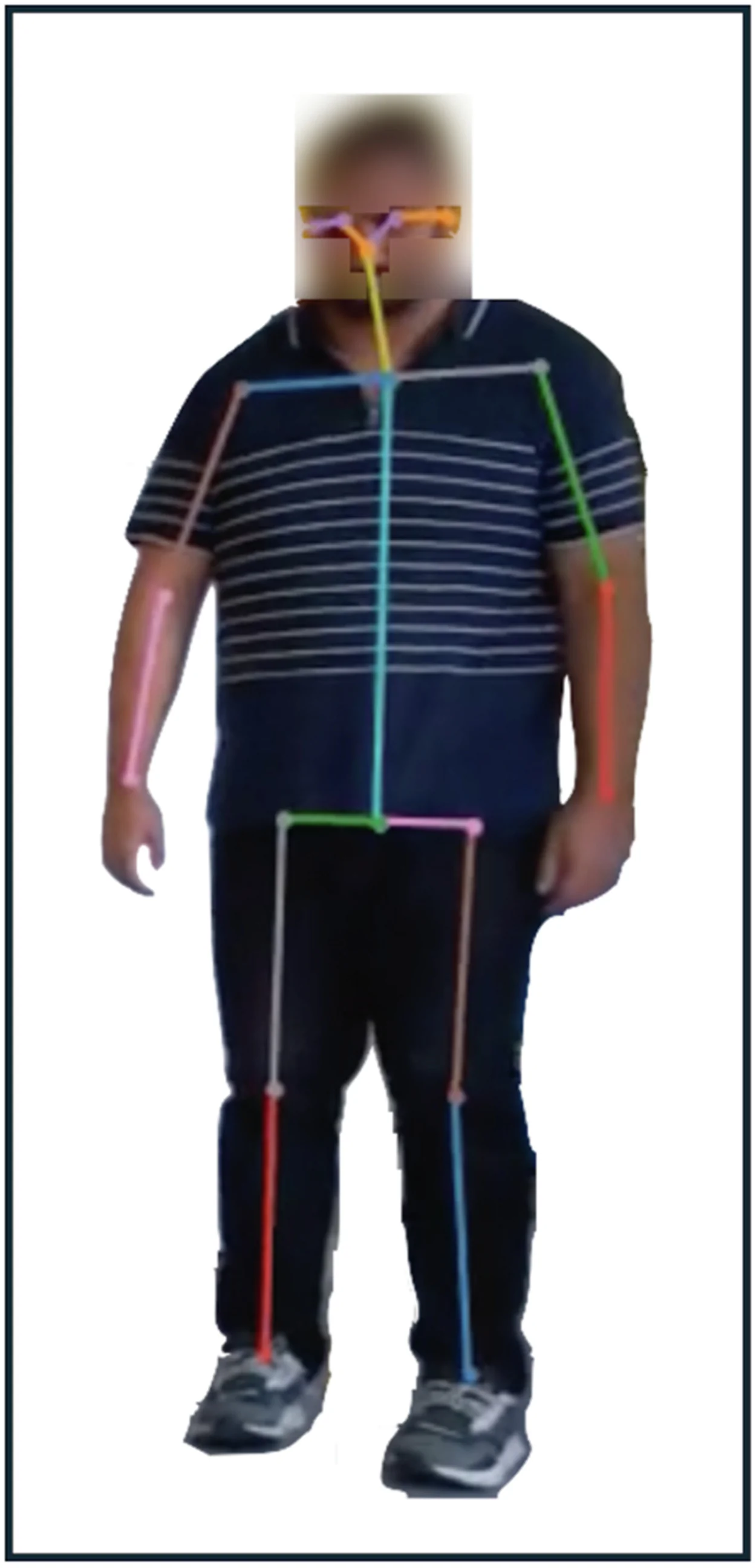
Example pose skeleton. Illustration of a skeleton representation generated by a human pose estimation model.

## Methods

This was an observational, cross-sectional proof-of-concept study using secondary analysis of video-recorded POMA.

### Participants

Healthy participants were adults with no self-reported neurologic or musculoskeletal conditions affecting gait or balance. Participants with stroke had a confirmed clinical diagnosis of stroke and were able to complete the POMA with or without supervision. Participants were excluded if they were unable to follow verbal instructions or safely attempt the assessment tasks. A total of 73 participants were involved across all the videos, with 56 participants without stroke and 17 with a history of an acute stroke within the last 8 weeks. Ethical approvals for both healthy participants and patients with stroke were obtained.

### Hardware

Using Microsoft Windows and the Microsoft Azure Kinect Software Development Kit, 2 cameras were externally synchronized, with the high-angle camera designated as the master and the hip-level (side-view) camera as the subordinate. Color video was recorded at 30 frames per second at a resolution of 1920 × 1080. We employed the hardware setup described by Mah et al.^22^

### Locations

Filming was conducted at King’s College Hospital National Health Service Foundation Trust, within their neurorehabilitation gym and stroke unit multipurpose room.

Two armless chairs were placed 5 m apart, facing each other. The high-angle camera was placed behind one chair, and the hip-level camera was positioned to the side of the second chair. The cameras were arranged to ensure that both chairs were within the field of view, and the entire body of the subject was in frame when standing in front of either chair.

### Balance and gait task (POMA)

The POMA was conducted by a trained therapist. The POMA is conducted as a structured, clinician-observed evaluation of a patient’s balance and gait during a series of everyday movements. It comprises 16 items scored using standardized behavioral descriptors to produce an ordinal score from 0 to 28, where lower scores indicate greater impairment. It is typically administered using a standard chair (with and without armrests) and a short walking space of several meters. The individual is observed while seated, then asked to stand, with the clinician assessing immediate balance, posture, and stability. Tasks include standing with eyes open, responding to a gentle nudge, turning 360°, and sitting down in a controlled manner. In the gait section, the individual walks at a normal pace while the assessor evaluates gait initiation, step length and height, symmetry, continuity, path, trunk movement, and stance. Each component is scored using defined criteria based on observed performance.^17^ The assessment could be started at either chair, with the high-angle camera behind or in front of the patient. Participants were evenly distributed between the 2 options. This was to vary the perspective of the cameras with respect to the assessment and increase the diversity of video information, while keeping the assessments the same.

### Video clip processing

Each assessment was recorded as a continuous file, with 1 file per camera perspective. In this study, we used the Skinned Multi-Person Linear 24 body model, which describes the human pose using 24 key points (joints), based on major landmarks of the body.^23^ This skeleton model was chosen because it included functionally important joints while allowing a degree of computational efficiency. The key points of the participants were extracted for every frame of video using the Metric-Scale Truncation-Robust heatmaps for Absolute^12^ 3D human pose estimation model, and provided as coordinates in 3D space.

**Fig 2:**
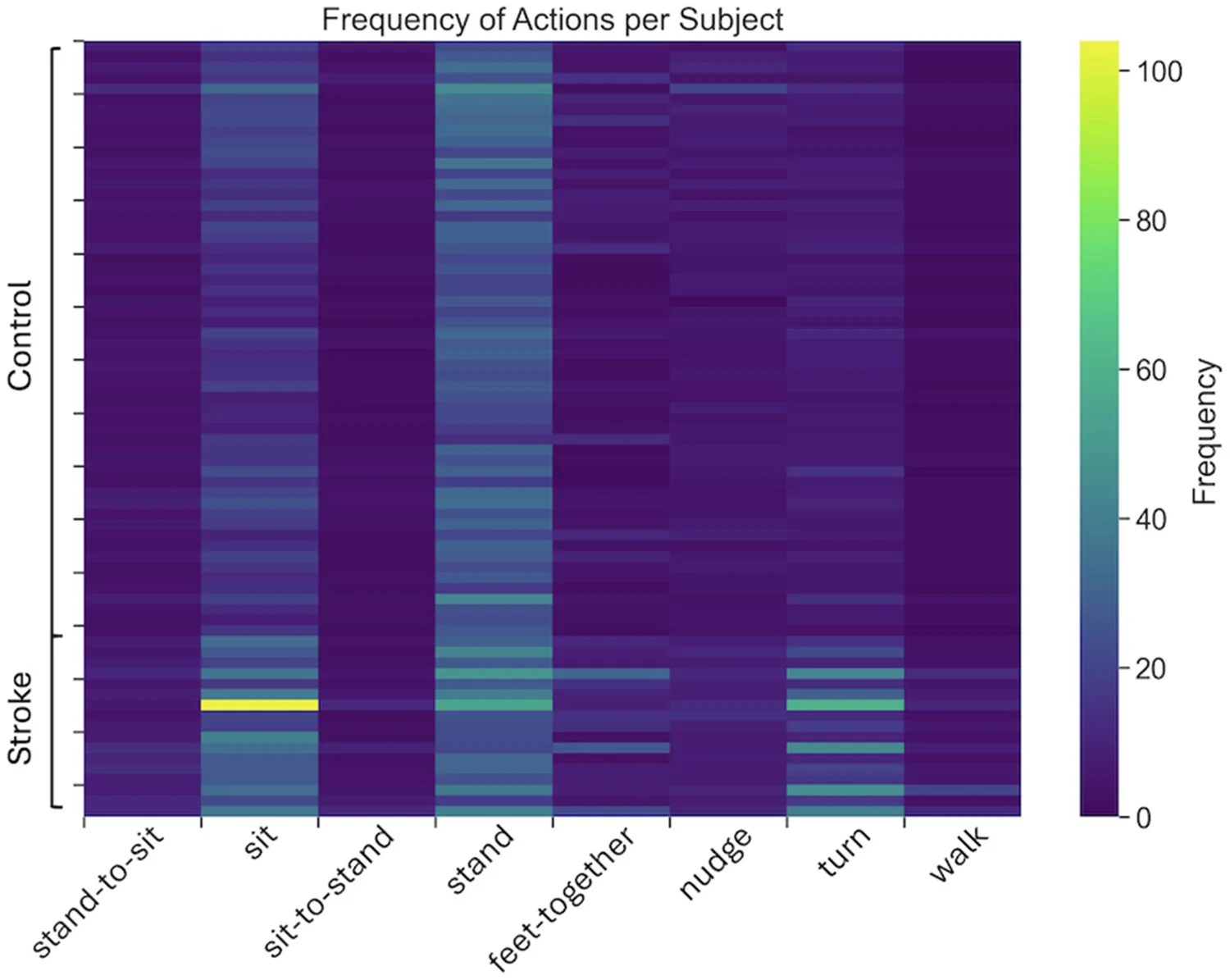
Distribution of clip frequency. Each POMA assessment was deconstructed into 1-second clips, with each clip assigned an action label based on the manual segmentation of the assessment. Actions that took longer to complete have a higher number of clips. Here we show the distribution of clip frequency for the different actions within the POMA assessment.

Each assessment video was manually reviewed by a trained clinician to label the different actions performed within the POMA. Figure 2 shows the different assessment sections, along with the total number of frames for each action type.

### Pose analysis

For analytic purposes, items from the POMA were clustered into broader functional categories. All gait-related items were grouped under “walk.” Within the balance domain, “attempts to rise” and “rises from a chair” were combined into the category “stand-to-sit,” whereas “standing balance” and “immediate standing balance” were grouped under “stand.” The remaining balance items were retained as individual categories. This clustering allowed the model to evaluate movement at the level of functional synergies rather than isolated tasks.

**Fig 3:**
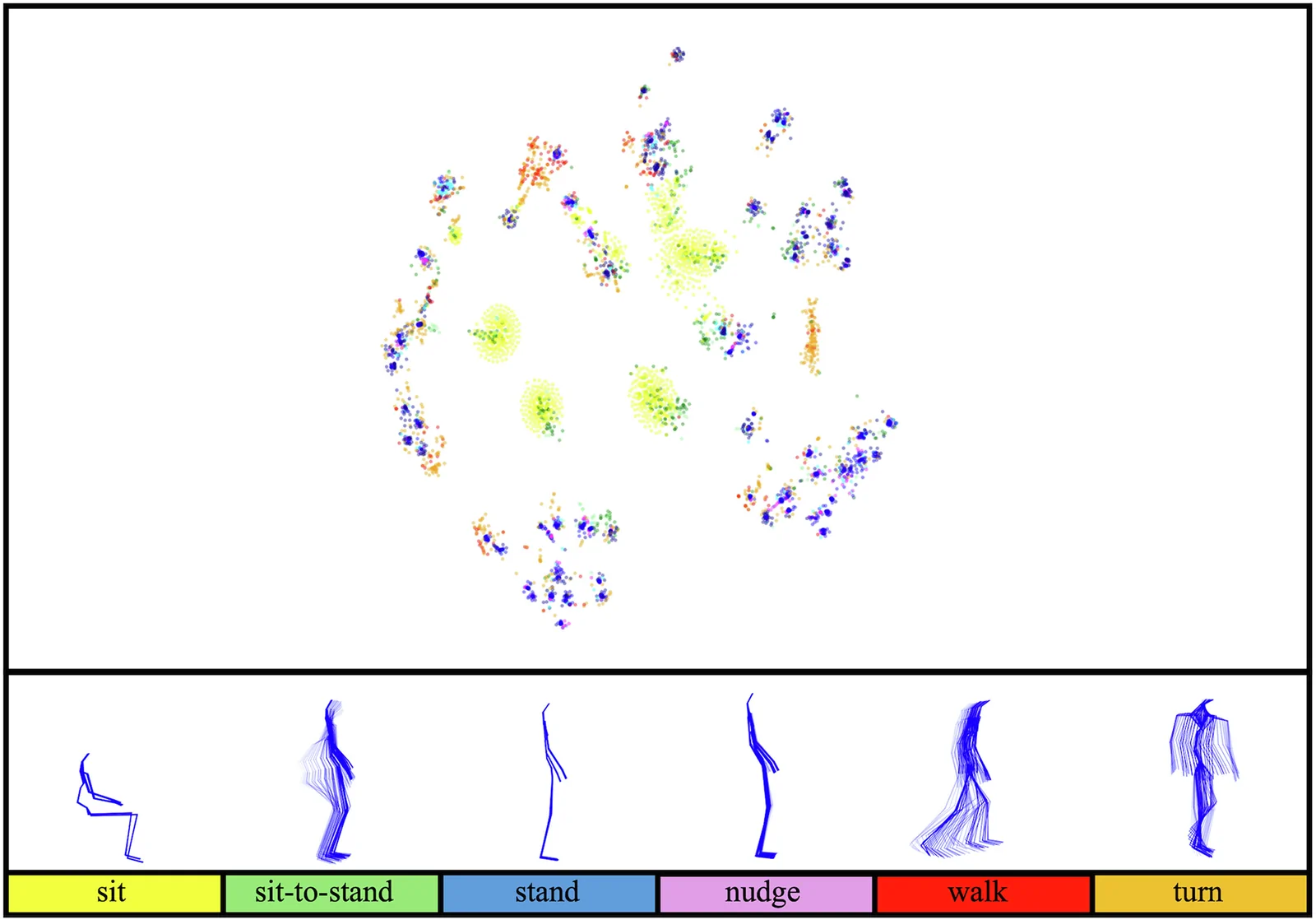
Task clip embedding. 2D embedding of 30-frame video clips using conditional t-SNE. Each data point represents a 2-second video clip colored by the manually labeled action performed within the POMA assessment. The greater the similarity between data points, when assessed by the pattern of key points within the 30-frame clip, the closer the data points are plotted together.

The Skinned Multi-Person Linear 24 body model defines the pelvis as the root key point, with all other key points specified relative to this location. This enables comparisons across different participants while accounting for variations in perspective, body size, and zoom. Each video was segmented into 30-frame clips, yielding 6740 clips of 1-second duration. The key points extracted from each clip were then passed to a dimensionality reduction algorithm, conditional t-distributed Stochastic Neighbor Embedding (t-SNE),^24^^,^^25^ to embed the high-dimensional data into a two-dimensional (2D) space. This provides a visual means of examining how the algorithm structures the data. Each data point represents a single 30-frame clip, positioned according to the similarity of its underlying keypoint patterns, such that more similar clips are plotted closer together. Data points were colored according to manually assigned action labels (fig 3).

These clips were subsequently used to train a classification algorithm, eXtreme Gradient Boosting (XGBoost),^26^ to assign each clip to an action category (eg, walking, standing). The classifier uses skeleton information from every frame within a clip to inform this decision and is optimized to maximize classification accuracy on labeled data. Data were split into training and test sets (80/20 split, random seed=42), with an additional unseen validation set comprising 2 POMA assessments used to assess overfitting. Within the training set, a leave-one-subject-out cross-validation approach was employed to reduce within-subject dependency. The model was trained using 500 trees, a learning rate of 0.01, and a maximum tree depth of 15, with optimization based on log loss. Model performance was evaluated using precision, recall, and F1 score. Precision quantifies the proportion of trajectories predicted as a given action that were correctly classified, whereas recall (sensitivity) reflects the proportion of true instances of that action detected by the model. The F1 score provides a balanced summary of precision and recall for each POMA action. For example, for sit-to-stand, precision indicates how many predicted sit-to-stand trajectories were correct, whereas recall indicates how many true sit-to-stand trajectories were detected.

## Results

### Demographics

A total of 73 subjects were recorded, with 56 controls and 17 patients with stroke. The mean ages were 37.8 years (SD 13.9, age range 20-76), and 68.9 years (SD 12.8, age range 44-85), respectively.

### Action recognition

The number of action clips is important for model training because it influences the range of movement patterns learned. Figure 2 shows the total clips per action. Standing, sitting, and turning were most frequently represented, whereas rising from a chair, walking, and standing feet together were least represented. Participants with stroke occupied the lower rows and generally contributed more clips than controls, particularly for turning, reflecting longer task durations. Figure 3 presents a 2D embedding of all 1-second clips, with sitting actions clustered near the center and standing actions distributed more peripherally.

### Accuracy of action recognition

The XGBoost model achieved a mean action classification accuracy of 78.9% (3 significant figures). Table 1 reports performance metrics for each action in the training set. The model performed best for sitting, standing, and turning, with F1 scores of 0.95, 0.82, and 0.80, respectively. Standing feet together and walking were the most challenging actions, with F1 scores of 0.34 and 0.42, respectively. Performance on the independent validation set is shown in table 2.

**Table 1:** Performance metrics on the training dataset

Class	Precision Mean (SD)	Recall Mean (SD)	F1 Score Mean (SD)
Stand-to-sit	0.88 (0.03)	0.72 (0.04)	0.79 (0.02)
Sit	0.92 (0.01)	0.98 (0.01)	0.95 (0.01)
Sit-to-stand	0.93 (0.04)	0.60 (0.06)	0.73 (0.06)
Stand	0.73 (0.01)	0.94 (0.01)	0.82 (0.01)
Feet-together	0.74 (0.10)	0.22 (0.06)	0.34 (0.07)
Nudge	0.68 (0.03)	0.36 (0.04)	0.47 (0.04)
Turn	0.74 (0.02)	0.89 (0.01)	0.80 (0.01)
Walk	0.68 (0.04)	0.31 (0.05)	0.42 (0.05)

NOTE. Performance metrics for the classification of video clips into specific actions within the POMA using XGBoost. A 10-fold leave-one-out cross-validation technique was used on the training data.

**Table 2:** Performance metrics on the “hold-out” dataset

Class	Precision Mean	Recall Mean	F1 Score Mean
Stand-to-sit	1.00	0.86	0.92
Sit	0.91	1.00	0.95
Sit-to-stand	1.00	0.67	0.80
Stand	0.70	0.91	0.80
Feet-together	0.50	0.20	0.29
Nudge	0.29	0.17	0.21
Turn	1.00	0.94	0.97
Walk	1.00	0.75	0.86

NOTE. Performance metrics for the classification of video clips into specific actions within the POMA using XGBoost. The trained model was assessed on 2 unseen assessments.

### Action quality

**Fig 4:**
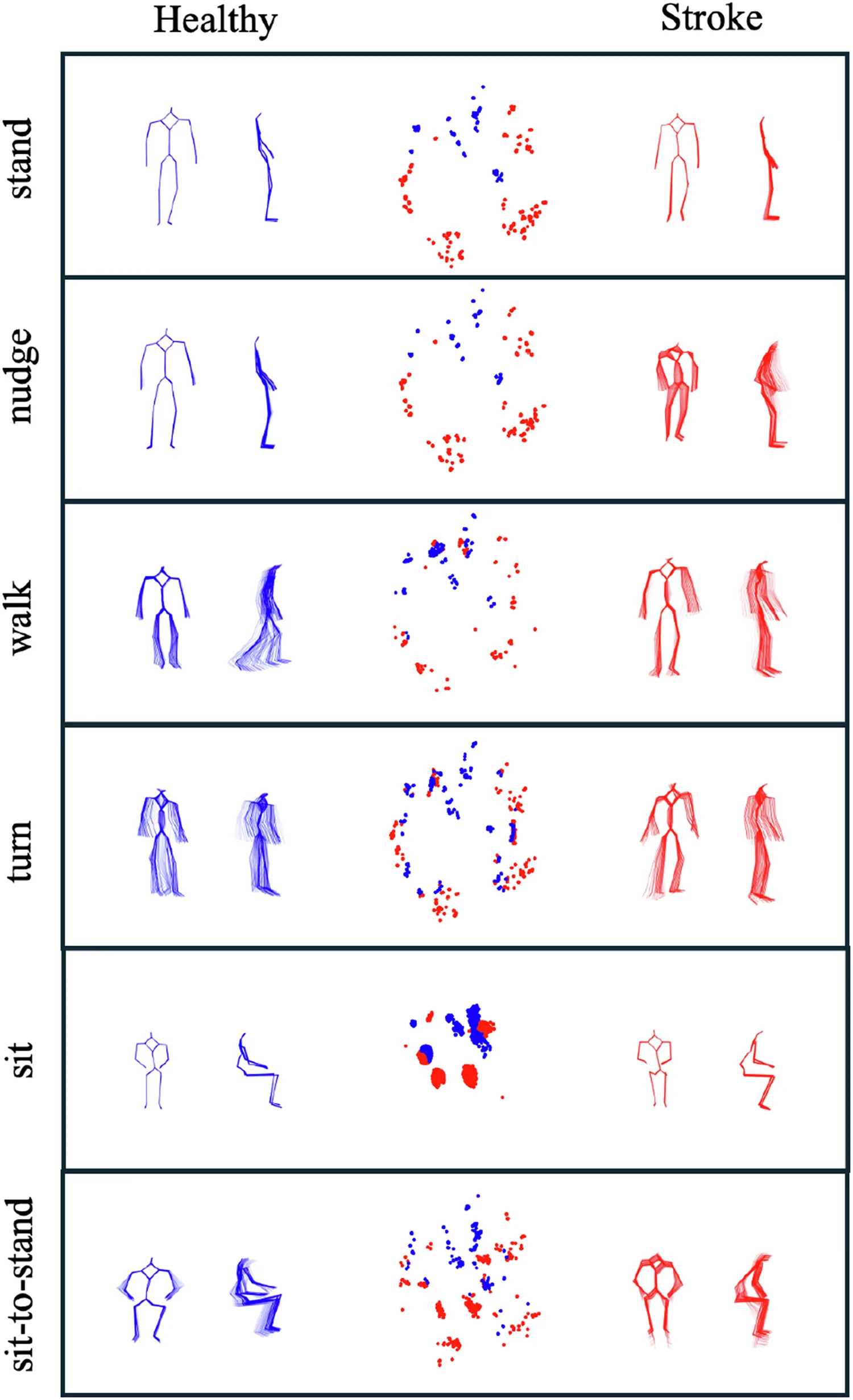
Task clip skeletons. 2D embedding of data isolated by specific action and colored by diagnosis state. Example overlay of skeleton poses from a single clip (30 skeletons) with darker lines indicating poses later in the sequence (left: healthy; right: stroke).

Figure 4 shows action embedding colored by participant group (healthy [blue] vs stroke [red]). Supplemental figure S1 (available online only at http://www.archives-pmr.org/) displays the same embedding pattern, but with the data points colored by summed POMA item score. Appendix 1 details the items contributing to each action score. For standing and nudge, healthy participants with higher scores cluster along a northwest–southeast axis, whereas lower scores lie more peripherally. In walking, lower POMA scores shift away from this axis toward the periphery, suggesting greater pose similarity between standing, nudge, and walking. Good turning performance is also more peripheral. For sit-to-stand, healthy participants cluster centrally, whereas participants with stroke are more dispersed.

## Discussion

Although the repertoire of human movement is extensive, many everyday actions performed by healthy individuals display consistent, recognizable patterns. Here we trained a pose estimation model on healthy participants and applied it to stroke survivors assessed with the POMA. Despite heterogeneity in lesion characteristics, stroke produces stereotyped motor changes; for this reason, impairment measures require validation in the target clinical population to ensure they capture clinically meaningful deviations.

We employed Metric-Scale Truncation-Robust Heatmaps for Absolute 3D Human Pose Estimation, an open-source 3D human pose estimation framework that infers the spatial location of major joints from color video. The reported joint localization accuracy of this system is approximately 40-80 mm. Although the model has been trained on millions of pose examples, it has not been fine-tuned for medical or rehabilitation-specific contexts. Although such spatial error limits its suitability for fine-scale biomechanical analysis, the accuracy is sufficient for higher-level applications such as action recognition and gross movement characterization, where millimeter precision is not essential.^27^, ^28^, ^29^

From the estimated joint positions, participant poses were reconstructed for each video frame, enabling visualization and analysis of movement trajectories across time. Figure 4 illustrates the superimposition of 30 consecutive poses from a single 1-second clip, with increasing opacity indicating temporal progression. Despite the limitations in joint resolution, clear qualitative differences between healthy participants and stroke survivors were evident across several POMA actions. During standing, participants with stroke demonstrated greater anterior–posterior sway than participants without stroke. Stroke frequently affects motor, proprioception, and visual control necessary for postural stability and is associated with asymmetric weight-bearing and reduced anticipatory postural adjustments.^30^ The increased sway observed here, which was also pronounced when participants were subjected to a nudge, is consistent with these impairments in sensorimotor function.^30^ During sit-to-stand transitions, healthy participants typically adopted a near-upright posture prior to rising, whereas participants with stroke showed increased forward trunk flexion, positioning the shoulders over the knees. Successful sit-to-stand requires sufficient quadriceps strength and ankle dorsiflexion on both limbs and core stability to generate the momentum needed for vertical rise. Hemiparesis after stroke reduces force production capacity in the affected limb,^31^ and increased trunk flexion represents a documented compensatory strategy to shift the center of mass anteriorly to help increase stability at the seat off stage.^32^ Indeed, in some instances, this approach is encouraged as a component of rehabilitation training.^33^

Differences in action quality were also reflected in the 2D t-SNE embedding of movement trajectories. Because t-SNE positions data points with similar high-dimensional features closer together, local clustering patterns derived from whole-body interjoint dynamics suggest that the trajectories encode features distinguishing both POMA actions and aspects of movement quality between controls and participants with stroke. However, spatial dispersion in this space should not be interpreted as direct variance in the original kinematic data, because t-SNE preserves local neighborhoods rather than global distances. In healthy participants, standing-related actions aligned closely along a linear axis, suggesting consistent postural control. In contrast, participants with stroke showed greater dispersion, with standing actions overlapping those associated with perturbation and turning, suggesting reduced static postural stability and greater reliance on compensatory strategies. These clinically meaningful differences indicate that relatively simple models can capture relevant control–stroke distinctions, whereas more complex models may exploit additional latent movement features.

To evaluate automated action recognition, we trained an XGBoost model to classify POMA-related actions from 1-second video clips. Performance varied across actions, with F1 scores ranging from 0.95 for sitting to 0.34 for standing feet together. Validation performance generally mirrored training performance, indicating limited overfitting and reasonable generalization. Walking was an exception, with an F1 score of 0.42 during training and 0.86 on the hold-out set. Because walking is dynamic, variable, and visually similar to other actions, the model may have struggled with both recall (detecting walking instances) and precision (avoiding misclassification) in the small training dataset. The higher hold-out performance is therefore likely due to sampling effects, making cross-validation a more reliable estimate of overall performance.

The reduced performance observed for certain actions is likely attributable to the use of short, fixed-length clips that did not align with the true onset and offset of each task. As a result, many clips captured transitional movements between actions, with standing frequently serving as an intermediate posture. This limitation disproportionately affected brief actions such as nudge and standing feet together, for which fewer clips contained a clear and isolated representation of the target action. This effect is also evident in the 2D embedding (fig 3), where actions did not form discrete clusters but instead appeared dispersed along continuous trajectories. Notably, a clear transition was observed between sitting and sit-to-stand or stand-to-sit actions, whereas nudge and turning overlapped substantially with standing.

The clustering strategy adopted in this work reflects an effort to capture clinically meaningful movement synergies rather than isolated task elements. By consolidating related POMA items, such as walking-related actions, the model was able to focus on broader motor states that may better differentiate healthy populations and populations with stroke within the constraints of the dataset. This approach mitigated issues arising from data sparsity and class imbalance and provides a foundation for future work aimed at identifying salient kinematic features within these motor states.

The resulting embedding enables visual interpretation of how the model processes movement data, offering insight into which features of motion are most informative for distinguishing between healthy individuals and stroke survivors. Although the embedding is currently coarse, it provides therapists with a transparent, data-driven perspective on movement quality that complements clinical observation. This interpretability is critical for fostering trust and facilitating integration of AI-based tools into clinical practice.

### Study limitations

Several limitations warrant consideration. Sampling bias may have arisen from convenience recruitment, class imbalance, and the single-center setting. Although the hold-out validation suggested limited overfitting, larger samples are required to strengthen confidence in the findings. The absence of age-matched controls also limits interpretation of whether observed differences reflect stroke-related impairment, age-related change, or both. Older healthy participants may therefore appear stroke-like if their gait or balance differs from younger controls. However, this may still be informative where the aim is to identify deviation from young healthy normative performance. The key clinical distinction is whether such movement shows age-related abnormalities or reflects age-appropriate compensation; future studies should therefore include age-matched controls to distinguish these effects from stroke-specific abnormalities.

Measurement uncertainty may have arisen from pose estimation error and manual action labeling, although labeling by a trained clinician aimed to minimize misclassification. Classification used fixed 1-second clips, which did not always align with true action boundaries and may have reduced performance for brief or transitional tasks. Automated temporal segmentation of POMA actions may reduce blurring between adjacent movements and support larger labeled datasets. More advanced spatiotemporal models may further improve performance but generally require larger training datasets.

The decision to cluster POMA items reflected the small stroke cohort and predominance of healthy data. Although this enabled exploratory group comparisons, larger and more diverse stroke cohorts are needed for finer-grained, item-level analyses. Future multicenter studies with balanced recruitment across age, impairment severity, and clinical settings would support external validation, improve generalizability, and test whether the observed movement features remain robust across different populations and assessment environments. Nevertheless, this proof-of-concept work demonstrates the potential to identify movement features that deviate from expected healthy performance.

## Conclusions

Advances in computer vision and AI may support automated motor assessment in rehabilitation. Using the POMA as an exemplar, this proof-of-concept study suggests that open-source 3D pose estimation can reconstruct skeletal movement, recognize common clinical actions, and identify movement features that differ between healthy individuals and stroke survivors. These findings should be interpreted cautiously given the study limitations. Although current pose estimates cannot replace high-precision biomechanical assessment, they may provide a scalable and clinically interpretable approach to movement screening and characterization. Further development using longer temporal inputs, larger and more diverse datasets, and multicenter validation is required. Nevertheless, this work supports cautious optimism that affordable pose-based assessment could complement clinical evaluation and contribute to rehabilitation monitoring, documentation, and risk detection.

## Disclosure

A.E. holds shares in AMD. J.C. holds stocks in Hologen and Elaitra, and has been awarded grant funding from UKRI scholarships and the EU MSCA. P.N. is affiliated with Hologen, a UCL spin-out company in health care generative modeling, and has been awarded grant funding from the Wellcome Trust and the UCLH NIHR Biomedical Research Centre. The other authors have nothing to disclose.

## Appendix Supplementary materials

Image, application 1

Image, application 2

## Footnotes

Supplementary material associated with this article can be found, in the online version at doi:10.1016/j.arrct.2026.100673.
