# Supplementary material for: Toward Automation of Motor Assessments in Stroke: Proof-of-Concept Model to Interpret Balance and Gait in Patients With Stroke
Source: Arch Rehabil Res Clin Transl. 2026 Jul 12;8(3):100673. doi: 10.1016/j.arrct.2026.100673 (PMC13591176; doi:10.1016/j.arrct.2026.100673)
Supplement: Supplementary file 1 [file mmc2.pdf]

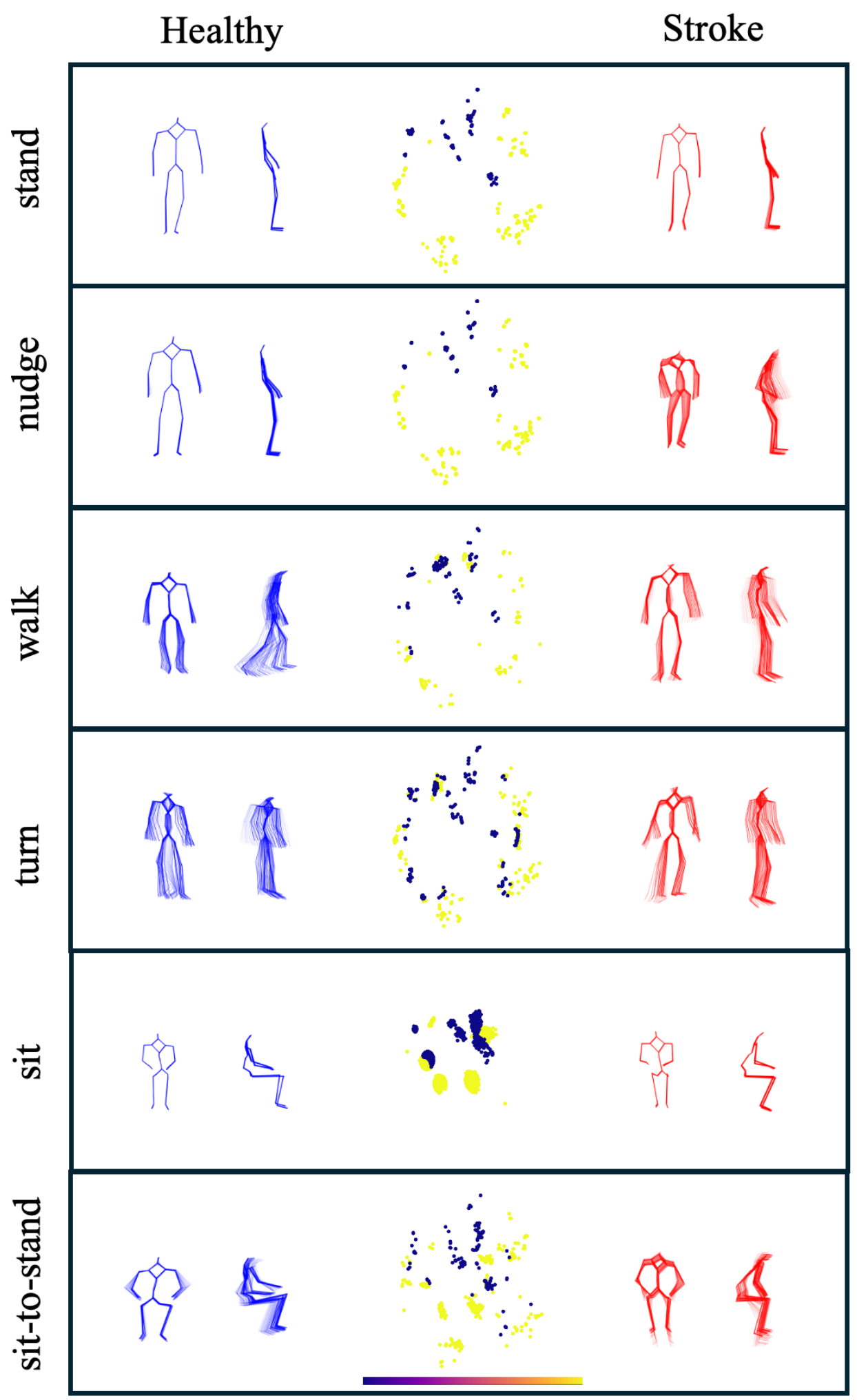

Healthy
Stroke
stand
nudge
walk
turn
sit
sit-to-stand
