# Supplementary material for: Toward Automation of Motor Assessments in Stroke: Proof-of-Concept Model to Interpret Balance and Gait in Patients With Stroke
Source: Arch Rehabil Res Clin Transl. 2026 Jul 12;8(3):100673. doi: 10.1016/j.arrct.2026.100673 (PMC13591176; doi:10.1016/j.arrct.2026.100673)
Supplement: Supplementary file 2 [file mmc1.docx]

Appendix 1

Balance section

| Sitting balance | Sit |
| --- | --- |
| Arising | Sit-to-stand |
| Attempts to arise immediate standing balance | Sit-to-stand |
| Standing balance | Stand |
| Nudge test | Nudge |
| Eyes closed | Feet-together |
| Turn 360° | Turn |
| Sitting down | Stand-to-sit |

Gait section

| Initiation of gait | Walk |
| --- | --- |
| Step length and height | Walk |
| Step symmetry | Walk |
| Step continuity | Walk |
| Path | Walk |
| Trunk sway | Walk |
| Walking stance | Walk |
